# The impact of different researchers to capture quality of life measures in a dementia randomised controlled trial

**DOI:** 10.1186/s13063-022-07064-4

**Published:** 2023-01-17

**Authors:** Rachel Evans, Paul Brocklehurst, Jean Ryan, Zoë Hoare

**Affiliations:** NWORTH Clinical Trials Unit, Bangor University, Holyhead Road, Bangor, Gwynedd LL57 2PZ UK

**Keywords:** Randomised controlled trial, Researcher effect, Researcher continuity, Bias, Outcome measurement, Quality of life, Dementia

## Abstract

**Background:**

Capturing changes in health and wellbeing within randomised controlled trials (RCTs) can be complex. The precision and accuracy of outcome scales to measure change is crucial, and therefore, consideration needs to be given to potential measurement errors when collecting these outcomes.

Many RCTs use multiple researchers to collect data, which has the potential to introduce variation in measurements. This study aimed to identify if there was a measurable effect of using different researchers to collect repeated assessments of quality of life (QoL) at different time points.

**Methods:**

A previously conducted study assessing the impact of reminiscence therapy on participants with dementia and carer (PwD-carer) dyads, ‘REMCARE’ (Reminiscence groups for people with dementia and their family caregivers), provided the platform for this exploratory secondary analysis. Data was categorised into two broad groups: those where the same researcher attended all assessments and those where different researchers undertook the assessments. ANCOVA (analysis of covariance) models used in the original REMCARE analysis with the addition of the ‘researcher-continuity’ variable were run on two QoL measures, the QoL-AD (Quality of Life in Alzheimer’s Disease) and QCPR (Quality of the Caregiving Relationship).

**Results:**

Three hundred thirty PwD-carer dyads were included in the analysis. For the PwD, a statistically significant effect was found on the researcher continuity variable for the QoL-AD and QCPR outcome measures at follow-up 1 but not at follow-up 2 signifying an impact of researcher attendance at the first follow-up but not follow-up 2. For the carer data, analyses revealed no statistically significant effects at follow-up 1; however, the QoL-AD measure at follow-up 2 was found to be statistically significant.

**Conclusions:**

These exploratory results indicate the possible impact of researcher continuity on QoL outcomes in dementia studies. Further research is required to explore this further and establish causality. If demonstrated, this would have implications for the planning of future empirical studies in dementia, in order to reduce this potential source of bias.

**Supplementary Information:**

The online version contains supplementary material available at 10.1186/s13063-022-07064-4.

## Background

There is potential to introduce bias when collecting and evaluating outcomes for randomised controlled trials (RCTs) [21, 27]. Dementia trials that focus on improving the quality of life (QoL) of participants are particularly susceptible to measurement bias due to the characteristics and symptoms of the disease and the potentially subjective nature of some of the measures used. Dementia is a broad umbrella term for a group of diseases characterised as “different brain disorders that trigger a loss of brain function” [9, 32]. Whilst the severity of the condition advances at different rates, the disease is progressive and eventually leads to death [32]. Whilst different dementia disorders share similar characteristics, the symptoms of the diseases vary on a case-by-case basis [2]. In general, patients tend to suffer with memory loss, confusion, reduced understanding, difficulties learning new tasks and issues conducting daily activities [1, 32, 33]. This typically impacts on the QoL for both dementia patients and their carers [7, 17]. QoL is described as “the standard of health, comfort and happiness experienced by an individual or group” ([23]). QoL has become an important consideration for dementia research over the last few decades with interventions to improve QoL being increasingly examined [19, 24]. Thus, the measurement of QoL in dementia has become a key focus to evaluate these interventions [20, 30].

Several studies have demonstrated that dementia-specific QoL measures appear to be reliable [8, 24]. However, less attention has been paid to the interview process itself and whether the use of different researchers may have an impact on QoL measurement over the duration of a trial [5, 16, 21, 27, 29]. Although research is available on the impact of researchers on participants’ responses [5, 29], there is little evidence on the impact that researcher continuity has on outcome data collection between visits. Kobak [16] suggests that researcher consistency may introduce potential biases, given the prior knowledge and/or relationship that the researcher may have with the participant over the course of a trial. This is particularly relevant for QoL trials with dementia patients, given the number and nature of the data collection (commonly through interviews).

The impact of using multiple raters across multiple time points has received limited attention and is influenced by the prevailing philosophical stance of some trialists, based on positivism and empiricism, where there is an assumption that a measure remains reliable over time [6, 12, 22]. Given the nature of dementia, QoL and the potential for rapport to develop over multiple time points (a method commonly used in QoL trials in dementia), this assumption requires testing. The researcher-participant relationship and the effects of rapport on interviews and assessments is discussed widely in the context of the qualitative paradigm [3, 12, 22], yet its impact on outcome measurement in trials is less understood. We hypothesised that the researcher- participant relationship could have the potential to influence the outcome measure. The participant with dementia measures were captured from the individual themselves with interactions from the researcher it was therefore thought that the measure responses could potentially be influenced by this interaction. Carer measures were provided independently, and therefore, it was hypothesised as unlikely to be influenced by the researcher relationship. Using these two perspectives of a single measure, we were aiming to elicit whether there was a researcher relationship effect. This explorative study aimed to identify if there was a measurable effect of using different researchers to collect repeated assessments of quality of life (QoL) at different time points.

## Methods

Data from a previously conducted RCT—the REMCARE study ‘Reminiscence groups for people with dementia and their family caregivers – effectiveness and cost-effectiveness pragmatic multi centre randomised trial’—was used [31]. This was a multi-centre parallel two-arm study, run across the United Kingdom (UK) (London, Manchester, Bangor, Hull, Bradford and Gwent) which aimed to test joint reminiscence groups for people with mild to moderate dementia and their family carer (relative or other carer who could act as an informant [31]). Those recruited and consented were randomised on a 1:1 allocation ratio to either the treatment arm or the treatment as usual. The participants were stratified by recruitment centre and their relationship to the carer (horizontal or vertical; horizontal being a carer of the same age or generation (e.g. a spouse, friend or sibling), whilst vertical is a different generation (e.g. parent and child relationship)) [31]. The main aim of the intervention was to improve the quality of life (QoL) for the person with dementia (PwD) and to reduce carer-related stress for the caregiver, subsequently improving the participant-carer relationship. However, the study showed no evidence for the effectiveness or cost-effectiveness for the intervention [31].

This study focuses on two REMCARE outcome measures that were completed by both the PwD and the carer: the Quality of the Caregiving Relationship (QCPR) [26], a validated measure used to assess the relationship between a participant and their carer by evaluating the presence of warmth in their relationship and the absence of conflict and criticism, and the Quality of Life in Alzheimer’s Disease (QoL-AD) [18], a validated self-report outcome measure that is used to assess the QoL of a person with dementia. Both of these measures required interaction and communication between the PwD and researcher and therefore had the potential to be influenced, whilst the carer completed the measures alone.

A third QoL measure, the EQ5D was also considered however is a relatively short and generic measure, which can be independently completed by the participants with little interaction from the researcher; thus, the measure is unlikely to be susceptible to a researcher effect and therefore is not included for this analysis. The QCPR was collected from both the PwD and carer based on their own perceptions of the caregiving relationship. The participant QoL-AD was collected from the PwD based on their perception of their own QoL, and the carer QoL-AD proxy version was collected from the carer regarding their view of the QoL of the PwD they care for. It follows that an effect, if there is one, would be apparent within the PwD responses but not the carer responses.

In total, the REMCARE study recruited 488 dyads (participant-carer), and data was collected at baseline, 3 months after baseline (follow-up 1) and 10 months after baseline (follow-up 2). As this analysis is exploring a researcher effect across the time points, based on a relationship building hypothesis, only PwD that completed all three visits were used; this resulted in a dataset containing 336 participants (69% of the REMCARE sample). A further six participants were removed due to the carers not completing all three follow-ups. Therefore, the final data sets contained 330 participants in each totalling 660 (330 dyads—the PwD and their corresponding carer, 68% of the REMCARE sample).

A ‘researcher attendance’ variable was created for inclusion in the models to represent researcher continuity. PwD’s were categorised into ‘researcher attendance’ groups based on whether they had the same researcher for all visits, different researchers or a combination. At follow-up 1, only two visits had been completed therefore participants were dichotomised into either the “same researcher” or “different researcher” collecting baseline data to the follow-up 1 data. At follow-up 2, three visits had been completed; therefore, participants were put into one of three categories: *one* researcher attending all three visits to collect data (i.e. “same researcher”), *two* visits with the same researcher but one of the visits was completed with a different researcher to the other two visits (i.e. “one same and two different researchers”) or lastly, *three* different researchers attending the visits therefore had all different researchers collecting outcome data (i.e. “different researchers”).

Analysis of covariance (ANCOVA) models were used at follow-up 1 (3 months) and follow-up 2 (10 months) to evaluate the impact of the researcher attendance variable, using the corresponding analysis models used in REMCARE, which accommodated for Age, Gender, Marital Status, Centre (study site), Wave (recruitment phase within centre) and Allocation. The carer model also included PwD Age and PwD Gender. In total, eight separate models were run, across the two outcomes, QoL-AD and QCPR, at the two follow-up time points for both PwD and carer data.

Sensitivity analysis was also conducted on assumptions about the consistency of researcher attendance for the second follow-up. The theory was that consistency in attendance of the researcher may build-up rapport with the PwD, potentially impacting the outcome scores. In the scenario where there were three visits by two researchers, this was either achieved with consistency for two visits with either the first or third conducted by another researcher, or, the first and third visit conducted by the same researcher and another researcher attending the ‘middle’ second visit. The consistency of visiting was broken up within the second scenario here and so could be considered as three different researchers given the timespan covered. Therefore, these visits where re-categorised as ‘three different researchers’ for the sensitivity analysis.

All data extraction, merging and subsequent analysis was conducted using SPSS IBM version 25 [15].

## Results

Table 1 details the frequencies of the researcher attendance at each follow-up along with the descriptive statistics for both PwD’s and carers presented overall and split by researcher attendance. Three hundred thirty dyads were included in the analysis. At follow-up 1, 160 (48%) participants had the same researcher and 170 (52%) had different researchers. By follow-up 2, 118 (36%) had the same, 129 (39%) had two and 83 (25%) had seen three different researchers. A large majority of the sample are White (PwD; 97%, carers; 96%), married (PwD; 75%, carers; 88%) and live with their spouse (PwD; 70%, carers; 79%). The prevalence of males and females in the data is almost equal for PwDs (male; 51%, female; 49%) but varies more for carers with a higher number of females (male; 33%, female; 67%). Some of the categories within the variables have low representation but the proportions across the researcher groups (same or different researcher(s)) within each variable level appear to be adequately split, except for the centre variable. The average age of the PwD is approximately 77 years old and for the carers 69 years old, with age ranges varying (PwD; 54–93, carers; 23–90). The mean ages between the researcher attendance groups are relatively similar.

**Table 1:** Descriptive statistics of demographics and other characteristics

Variable		***N*** = 330 ***N*** (%)
**Researcher attendance at follow-up 1**	Same researcher	160 (48%)
	Different researcher	170 (52%)
**Researcher attendance at follow-up 2**	Same researcher	118 (36%)
	Two different	129 (39%)
	Three different	83 (25%)
**PwD gender**	Female	162 (49%)
	Male	167 (51%)
	Missing	1 (< 1%)
**PwD ethnicity**	White	319 (97%)
	Other	10 (3%)
	Missing	1 (< 1%)
**PwD marital status**	Spousal	246 (75%)
	Non-spousal	82 (25%)
	Missing	2 (< 1%)
**PwD lives with**	No-one	49 (15%)
	Other	13 (4%)
	Other family	26 (8%)
	Spouse	229 (70%)
	Spouse and other family	11 (3%)
	Missing	2 (< 1%)
**Carer gender**	Female	221 (67%)
	Male	108 (33%)
	Missing	1 (< 1%)
**Carer ethnicity**	White	317 (96%)
	Other	10 (3%)
	Missing	3 (1%)
**Carer marital status**	Spousal	290 (88%)
	Non-spousal	36 (11%)
	Missing	4 (1%)
**Carer lives with**	No-one	10 (3%)
	Other	14 (4%)
	Other family	26 (8%)
	Spouse	260 (79%)
	Spouse and other family	15 (5%)
	Other and no one	1 (< 1%)
	Missing	4 (1%)
**Age**	Pwd age	*77 (7.60)
	Carer age	*69 (11.38)

*Mean (SD)

Descriptive statistics of the outcome measures at each time point are displayed in Table 2, presented overall and by researcher attendance groups. Overall, the completion rates of the outcome measures are high and only vary by a small percentage across time points. When split by researcher attendance variables the completion rates vary slightly in each group but still remain high (over 79%). A visual inspection of the completion rates indicates that there appears to be no major differences between the same or different researchers across time points, raising no concerns for analysis. For both measures, higher scores indicate a better result in terms of outcome measure results; for the QCPR, a better perceived relationship between the dyads and for the QoL-AD a better quality of life. In general, mean scores on both outcome measures decrease slightly across time points and overall, the carers have lower scores on both measures compared with the participant scores. The splits between the researcher attendance groups at the mean scores are generally evenly distributed across groups.

**Table 2:** Descriptive statistics of the outcome measures

Outcome measure	Overall		Researcher attendance at follow-up 1				Researcher attendance at follow-up 2					
			***Same*** ***N*** ***= 160***		***Two*** ***N*** ***= 170***		***Same*** ***N*** ***= 118***		***Two*** ***N*** ***= 129***		***Three*** ***N*** ***= 83***	
	***N*** (%)	Mean (SD)	***N***	Mean (SD)	***N***	Mean (SD)	***N***	Mean (SD)	***N***	Mean (SD)	***N***	Mean (SD)
**Participant—Qol-AD total**												
***Baseline***	**317 (96%)**	**37.5 (5.18)**	150	37.3 (5.42)	167	37.7 (4.97)	109	37.2 (5.36)	126	37.5 (5.18)	82	38.0 (4.97)
***Follow-up 1***	**309 (94%)**	**37.1 (5.71)**	145	36.8 (6.11)	164	37.2 (5.36)	106	36.4 (6.33)	123	37.6 (5.28)	80	37.0 (5.50)
***Follow-up 2***	**292 (89%)**	**36.4 (5.48)**	N/A				99	35.5 (5.48)	118	37.2 (5.05)	75	36.5 (6.03)
**Participant—QCPR total**												
***Baseline***	**308 (93%)**	**58.3 (6.04)**	149	57.9 (6.43)	159	58.7 (5.65)	108	57.3 (5.77)	124	60.0 (6.20)	76	58.6 (6.07)
***Follow-up 1***	**293 (89%)**	**58.1 (6.62)**	138	58.2 (6.82)	155	58.1 (6.45)	103	57.9 (6.65)	117	58.4 (6.74)	73	58.0 (6.45)
***Follow-up 2***	**288 (87%)**	**57.3 (6.47)**	N/A				98	57.4 (6.41)	114	56.6 (6.09)	76	58.2 (7.05)
**Carer—proxy Qol-AD total**												
***Baseline***	**326 (99%)**	**32.0 (6.13)**	157	31.8 (6.22)	169	32.1 (6.06)	115	31.0 (6.16)	129	33.0 (5.95)	82	31.7 (6.19)
***Follow-up 1***	**323 (98%)**	**31.1 (6.24)**	157	30.9 (6.31)	166	31.2 (6.18)	116	30.4 (6.25)	128	31.9 (6.29)	79	30.8 (6.08)
***Follow-up 2***	**329 (< 100%)**	**30.2 (6.06)**	N/A				118	29.7 (5.90)	129	30.7 (6.40)	82	30.1 (5.72)
**Carer QCPR total**												
***Baseline***	**321 (97%)**	**54.3 (8.58)**	156	53.7 (8.88)	165	55.0 (8.27)	115	53.0 (8.75)	128	55.5 (7.97)	78	54.3 (9.13)
***Follow-up 1***	**312 (95%)**	**53.6 (8.83)**	149	53.0 (9.29)	163	54.2 (8.37)	111	52.2 (8.94)	124	54.6 (8.41)	77	53.9 (9.18)
***Follow-up 2***	**321 (97%)**	**53.1 (9.56)**	N/A				117	52.2 (9.97)	126	53.0 (9.47)	78	54.6 (8.98)

*SD* standard deviation

Complete case analysis was conducted with no methods of missing data imputation adopted. Methods of dealing with missing data in RCTs is debated amongst researchers, although it is acknowledged that methods of dealing with missing data for analysis should be based on how much data is missing, the kind of missing data (single items, full measures, a measurement time point) and what type of missing data exists within the dataset (missing completely at random (MCAR), missing at random (MAR) or missing not at random (MNAR)). The REMCARE study adopted multiple imputation techniques for analysis. Since the completion rates of the outcomes to be used for the current analysis are high (between 87 and 99% see Table 2), the missing data should not affect the results of the current analysis. Scheffer [25] suggests complete cases can be used if no more than 6% of the data is missing, which in most cases of our outcomes (see Table 2) is the case. Predictors of ‘missingness’ were considered; however, no factors were deemed to predict missingness across the comparator groups indicating that the missing data should not cause bias in either comparator groups. It was therefore deemed not necessary to impute and complete case analysis was conducted with no methods of missing data imputation.

Model assumptions were checked. The assumption of homogeneity of regression slopes was violated in several cases. Where this assumption was violated, as recommended by Grace-Martin [10], the interaction term was included in the final model. The interaction terms that were significant and hence included in the main models are reported within the analysis results table. Where this occurs, the interpretation of the results is investigated further in relation to this interaction by assessing the relationships between the baseline and follow-up scores at each level [10].

### Model results

The results from the ANCOVA models are displayed in Table 3, and the adjusted estimated marginal means are presented in Table 4; the full model results can be found in Additional file 1. Where significance was indicated, then the associated effect sizes and confidence intervals are also presented (Additional file 1, Table S8). For all significant findings, pairwise comparison tests were conducted to assess the magnitude of the differences however none revealed significant results. For all significant findings in the models, there was an interaction term between the covariate and independent variable included due to the homogeneity assumption being violated. Therefore, the interpretation of these results are assessed in relation to the interactions. The R squared scores of the relationships between covariate from the significant interaction and the follow-up scores at each level are presented in Table 4.

**Table 3:** Results from ANCOVA models for researcher attendance variable

Analysis	Outcome Measure	Main effect			Interaction Term			
		DF	***F***-value	SIG	Term	DF	***F***-value	SIG
**PwD—main**	**QCPR fu 1**	1	5.65	^a^0.02	**QCPR B x Researcher Attendance**	1	6.00	^a^0.02
	**QoL-AD fu 1**	1	10.24	^b^< 0.01	**QoL-AD B x Researcher Attendance**	1	9.42	^b^< 0.01
	**QCPR fu 2**	2	2.93	0.06	**QCPR B x Researcher Attendance**	2	3.15	^a^0.05
	**QoL-AD fu 2**	2	1.14	0.32	N/A			
**PwD—sensitivity**	**QCPR fu 2**	2	4.02	^a^0.02	**QCPR B x Researcher Attendance**	2	4.35	^b^0.01
	**QoL-AD fu 2**	2	3.32	^a^0.04	**PwD Age x Researcher Attendance**	2	2.96	^a^0.05
**Carer—main**	**QCPR fu 1**	1	0.03	0.86	N/A			
	**QoL-AD fu 1**	1	0.70	0.40	N/A			
	**QCPR fu 2**	2	0.46	0.63	N/A			
	**QoL-AD proxy fu 2**	2	4.15	^a^0.02	**PwD age**^a^**Researcher Attendance**	2	3.96	^a^0.02
**Carer—sensitivity**	**QCPR fu 2**	2	1.26	0.29	N/A			
	**QoL-AD fu 2**	2	4.05	^a^0.02	**PwD age x Researcher Attendance**	2	3.98	^a^0.02

*SIG* significance, *FU* follow-up, *DF* degrees of freedom

^a^Significant at the 0.05 level

^b^Significant at the 0.01 level. *N/A* not applicable

**Table 4:** Adjusted values at each level of the researcher attendance variable from ANCOVA models

Analysis	Outome measure	Adjusted values at follow-up						R square values				
		Same researcher		Two different		Three different		Term	Overall	Researcher		
		***N***	Mean (SE)	***N***	Mean (SE)	***N***	Mean (SE)			Same	Two	Three
PwD—main	**QCPR fu 1**	132	58.0 (0.96)	144	57.9 (1.01)	N/A		**QCPR B**	0.31	0.49	0.16	N/A
	**QoL-AD fu 1**	136	37.2 (0.64)	161	37.8 (0.72)	N/A		**QoL-AD B**	0.43	0.59	0.29	N/A
	**QCPR fu 2**	92	58.0 (1.10)	108	57.3 (0.86)	70	57.8 (1.34)	N/A	N/A	N/A	N/A	N/A
	**QoL-AD fu 2**	91	36.7 (0.87)	115	38.0 (0.68)	74	37.5 (0.97)	N/A	N/A	N/A	N/A	N/A
PwD—sensitivity	**QCPR fu 2**	92	57.9 (1.11)	94	57.4 (0.90)	84	58.3 (1.24)	**QCPR B**	0.13	0.31	0.02	0.19
	**QoL-AD fu 2**	91	36.8 (0.87)	99	38.1 (0.70)	90	37.5 (0.88)	**PwD Age**	< 0.001	0.01	0.005	< 0.001
Carer**—**main	**QCPR fu 1**	145	52.7 (1.00)	156	52.9 (0.98)	N/A		N/A	N/A	N/A	N/A	N/A
	**QoL-AD proxy fu 1**	153	31.9 (0.67)	163	31.3 (0.67)	N/A		N/A	N/A	N/A	N/A	N/A
	**QCPR fu 2**	113	53.1 (1.46)	123	53.0 (1.13)	72	54.4 (1.65)	N/A	N/A	N/A	N/A	N/A
	**QoL-AD proxy fu 2**	114	31.7 (0.90)	127	30.9 (0.69)	79	31.8 (1.01)	**PwD age**	0.02	0.07	0.008	0.001
Carer**—**sensitivity	**QCPR fu 2**	113	53.1 (1.47)	107	52.4 (1.15)	88	54.5 (1.51)	N/A	N/A	N/A	N/A	N/A
	**QoL-AD proxy fu 2**	114	31.8 (0.90)	111	30.7 (0.70)	95	32.0 (0.92)	**PwD age**	0.02	0.07	0.007	0.006

*N/A* not applicable, *SE* standard error

### PwD

At follow-up 1, the researcher attendance variable is statistically significant at the 1% level on the PwD QoL-AD, *F*(1, 267) = 10.24, *p* < 0.01 (Table 3) and on the PwD QCPR at the 5% level *F*(1, 246) = 5.65, *p* = 0.02 (Table 3). Adjusted mean differences between the researcher attendance groups are not statistically significant at the 5% level (QoL-AD; *p* = 0.36 and QCPR; *p* = 0.95) (Table 4). Based on the R scores of the interaction association overall, the baseline scores have a positive relationship with the follow-up scores. When split by the researcher attendance groups, the relationship between baseline and follow-up scores is stronger in the same researcher group compared with the different researchers group.

At follow-up 2 for the primary analysis, the researcher attendance variable was not statistically significant on either the QoL-AD, *F*(2, 246) = 1.14, *p* = 0.32, nor on the QCPR, *F*(2, 234) = 2.93, *p* = 0.06 (Table 3). The sensitivity analysis of the re-coded variable, however, produced a significant finding at the 5% level on the PwD QoL-AD *F*(2, 280) = 3.32, *p* = 0.04 and on the PwD QCPR *F*(2, 233) = 4.02, *p* = 0.02 (Table 3). Similarly, to follow-up 1 results, the R squared scores indicate that there is a positive relationship between the baseline scores and the follow-up scores and that this relationship is strongest in the same researcher group compared with the two different researchers group and three different researcher group.

### Carer

Primary analyses on the carer data found that the variable is not statistically significant on the QoL-AD at follow-up 1, *F*(1, 270) = 0.03, *p* = 0.86, on the QCPR at follow-up 1 *F*(1, 285) = 0.70, *p* = 0.40 nor on the QCPR at follow-up 2, *F(*2, 272) = 0.46, *p* = 0.63 (Table 3). There is, however, a statistically significant result on the QoL-AD proxy at follow-up 2 at the 5% level *F*(2, 282) = 4.15, *p* = 0.02 (Table 3). Overall, there is a very weak negative association with PwD age and proxy QoL-AD scores. The same researcher group has a slightly stronger negative association whereas the two different researcher and three different researcher groups have a slightly weaker negative relationship.

The results from the sensitivity analysis on the two carer outcomes at follow-up 2 produced no different findings on the researcher attendance variable to the primary models, suggesting that the recoding of the researcher attendance variable had no effect on the carer data.

## Discussion

The primary analysis on the PwD data revealed a statistically significant difference between different researcher attendance patterns on two different QoL outcome measures at the first outcome measurement point, yet the second outcome point found no such difference. However, sensitivity analyses that accounted for a break in researcher continuity revealed a statistically significant effect of the researcher attendance variable on both measures at the second follow-up. The results of the carer models revealed that the researcher continuity was not statistically significant on the QCPR at either time point nor on the QoL-AD at follow-up 1. Taken together, these appear to suggest that there was a researcher continuity effect detected.

These results could be related to on-going cognitive decline in the PwD study population. It is possible that at the second follow-up appointment, participants may not have been able to recall the researcher they saw at baseline or follow-up 1 and any rapport built up in previous assessments may have been lost (Association, [1]; “How dementia progresses”, [15, 33]). The time between the assessments may have exacerbated this phenomenon. Equally, from a statistical perspective, the way the variables were constructed could have contributed to the effect not being seen at follow-up 2. There are more groups at follow-up 2 compared with follow-up 1 meaning that smaller samples are represented in each resulting in less statistical power and more potential for a type 1 error. A third explanation also relates to how the variables were constructed, which would be supported by the results of the sensitivity analysis. The re-coding process may have inadvertently produced a statistically significant finding, with the effect being stronger, when compared to follow-up 1. However, the results still appear to show that rapport was built over time.

The effect seen on the carer QoL-AD at follow-up 2 was unexpected in relation to the hypothesis. This suggests that although the carer completed the measure independently, they may still have been influenced by researcher continuity. However, several elements could have contributed to this result which cannot be measured here and the impact of researcher consistency on carer data should be further explored.

The impact of researcher continuity has received little attention in the context of trial-based research in dementia studies examining QoL. Kobak [16] hypothesised that ‘researcher bias’ may be more prevalent in those situations where the same researcher collects both baseline and follow-up measures. This appears to be supported by the findings in this study. Whilst this can be seen in QoL studies more broadly, this appears to be more prominent in cases where the same researcher undertakes both assessments [11, 14]. Indeed, a paper [3] highlighted the importance of establishing rapport in quantitative assessments to encourage participants to respond more openly and honestly. The findings in this study suggest that although this process would be described as ‘good practice’, it is not without its effect on potential measurement bias and that more research is needed.

The current research has several limitations. The analyses presented here were explorative in nature and the data collected was not designed to test the hypotheses, i.e. it was a post hoc analysis and was not conceptualised or conducted a priori. As a result, the study was not powered to detect the effect of researcher continuity. Although the splits between researcher attendance groups are relatively similar (Table 2), allocation of both participants and researchers were not randomised. Multiple researchers were used (*n* = 43), and the variability between these researchers was not calculated or accounted for. Equally, the reliability of researchers’ scores is not known, and individual researcher characteristics, such as age, training, experience, warmth and ability to establish rapport, were not taken into account (given the lack of data). There was no allocation concealment for participants in REMCARE. As a result, researchers may have become accidentally unblinded during the follow-up assessments. Post hoc assessment in the original study found that researchers ‘were indeed more likely to be correct than incorrect in the direction of their prediction’ [31]. Furthermore, the sample lacks diversity in race and marital status. In terms of an exploratory analysis around researcher relationship this may reflect cultural and generational relationships which is an important limitation of the study. Visit length was also not recorded which may have had an impact and may need to be accounted for in future studies.

This explorative study indicates a possible researcher continuity effect on the outcome measure scores of participants in one dementia QoL trial. This effect may be relevant to all clinical trials and settings or may only reflect an issue within dementia research, further investigation would be required in other trial settings to establish this. Whilst the results imply that there is a possible effect present, they are not definitive and subject to the limitations outlined above. It should be noted that this study was not designed nor powered to detect this signal and all results and findings should be interpreted with this knowledge. To definitively address this question, future research should be conducted with the research hypotheses framed and powered a priori. This could be undertaken as a Study Within A Trial (SWAT), which is a “self-contained research study that has been embedded within a host trial with the aim of evaluating or exploring alternative ways of delivering or organising a particular trial process” [28].

Furthermore, dementia studies may not be the most appropriate disease area to evaluate researcher continuity, given the inter-relationship between rapport and memory, which is more difficult to establish in dementia trials [1, 33]. If an effect is demonstrated through future studies, then implications for the conduct and design of future RCTs would need consideration to account for this bias. RCTs could incorporate this bias in one of two ways—either through the logistical aspects of the research design or by incorporating some researcher effect into the analysis model. However, logistical limitations to the former may make this difficult and complicate site visits and would depend on their relative size and structure. RCTs could be designed so that researchers collecting outcome data attend visits to the same participant where possible. This may be very difficult for larger studies with several sites and would depend on the size and structure of the involved sites. Aspects such as staff turn around, number of follow-ups, follow-up length and number of patients would determine whether this could be achieved.

## Conclusion

Dementia trials with QoL end-points may be influenced by researcher continuity. However, further empirical research is needed to explore this possible relationship definitely.

## Supplementary Information

**Additional file 1: Table S5.** ANCOVA Model Results for PwD Data. Table S6: ANCOVA Model Results for Carer Data. **Table S7.** ANCOVA Model Sensitivity Analysis Results at Follow-up 2. **Table S8.** Adjusted Means for Researcher Attendance Groups from PwD ANCOVA Model.

## Data Availability

The data that support the findings of this study are not available as restrictions apply to the use of these data, which were used under permission from the chief investigator for the current study, and so are not publicly available.

## Abbreviations

ANCOVA: Analysis of covariance
EBM: Evidence-based medicine
NWORTH: North Wales Organisation for Randomised Trials in Health
PwD: People with dementia
QCPR: Quality of the Caregiving Relationship
QoL: Quality of life
QoL-AD: Quality of Life in Alzheimer’s Disease
RCT: Randomised controlled trial
REMCARE: Reminiscence groups for people with dementia and their family caregivers
SWAT: Study within a Trial
WHELD: Well-Being and Health for People with Dementia
UK: United Kingdom
