# Supplementary material for: The impact of different researchers to capture quality of life measures in a dementia randomised controlled trial
Source: Trials. 2023 Jan 17;24:33. doi: 10.1186/s13063-022-07064-4 (PMC9843939; doi:10.1186/s13063-022-07064-4)
Supplement: Supplementary file 1 — Additional file 1: Table S5. ANCOVA Model Results for PwD Data. Table S6: ANCOVA Model Results for Carer Data. Table S7. ANCOVA Model Sensitivity Analysis Results at Follow-up 2. Table S8. Adjusted Means for Researcher Attendance Groups from PwD ANCOVA Model. [file 13063_2022_7064_MOESM1_ESM.docx]

**Supplementary material**

**Appendix 1 – Full ANCOVA model results tables**

***Table 5****:* *ANCOVA Model Results for PwD Data*

| **QCPR follow-up 1 – model 1** | | | | **QoL-AD follow-up 1 – model 2** | | | |
| --- | --- | --- | --- | --- | --- | --- | --- |
| **Factor** | **DF** | **F-value** | **p-value** | **Factor** | **DF** | **F-value** | **p-value** |
| **QCPR Baseline** | 1 | 104.5 | **<0.01 | **QoLAD Baseline** | 1 | 215.22 | **<0.01 |
| **Age** | 1 | 0.05 | 0.83 | **Age** | 1 | 1.28 | 0.26 |
| **Gender** | 1 | 0.74 | 0.40 | **Gender** | 1 | 2.59 | 0.11 |
| **Marital status** | 1 | 0.71 | 0.40 | **Marital status** | 1 | 0.02 | 0.89 |
| **Centre** | 6 | 0.34 | 0.92 | **Centre** | 6 | 1.78 | 0.10 |
| **Wave** | 4 | 2.27 | 0.06 | **Wave** | 4 | 0.49 | 0.74 |
| **Allocation** | 1 | 0.17 | 0.68 | **Allocation** | 1 | 0.92 | 0.34 |
| **Centre x Allocation** | 6 | 0.19 | 0.98 | **Centre x Allocation** | 6 | 2.22 | *0.04 |
| **Researcher Attendance** | 1 | 5.65 | *0.02 | **Researcher Attendance** | 1 | 10.24 | **<0.01 |
| **Centre x Researcher Attendance** | 6 | 1.35 | 0.23 | **Centre x Researcher Attendance** | 6 | 0.89 | 0.50 |
| **QCPR B x Researcher Attendance** | 1 | 6.00 | *0.02 | **QoLAD B x Researcher Attendance** | 1 | 9.42 | **<0.01 |
| **Error (SS within)** | 246 |  |  | **Error (SS within)** | 267 |  |  |
| **QCPR follow-up 2 – model 3** | | | | **QoL-AD follow-up 2 – model 4** | | | |
| **Factor** | **DF** | **F-value** | **p-value** | **Factor** | **DF** | **F-value** | **p-value** |
| **QCPR Baseline** | 1 | 43.28 | **<0.01 | **QoLAD Baseline** | 1 | 88.94 | **<0.01 |
| **Age** | 1 | 6.01 | *0.02 | **Age** | 1 | 0.45 | 0.50 |
| **Gender** | 1 | 0.00 | 0.99 | **Gender** | 1 | 0.29 | 0.60 |
| **Marital status** | 1 | 0.43 | 0.51 | **Marital status** | 1 | 0.86 | 0.36 |
| **Centre** | 6 | 0.85 | 0.53 | **Centre** | 6 | 2.50 | *0.02 |
| **Wave** | 4 | 0.53 | 0.71 | **Wave** | 4 | 1.14 | 0.34 |
| **Allocation** | 1 | 0.50 | 0.48 | **Allocation** | 1 | 0.27 | 0.60 |
| **Centre x Allocation** | 6 | 0.68 | 0.67 | **Centre x Allocation** | 6 | 0.83 | 0.55 |
| **Researcher Attendance** | 2 | 2.93 | 0.06 | **Researcher Attendance** | 2 | 1.14 | 0.32 |
| **Centre x Researcher Attendance** | 10 | 2.16 | *0.02 | **Centre x Researcher Attendance** | 10 | 0.74 | 0.69 |
| **QCPR B x Researcher Attendance** | 2 | 3.15 | *0.05 | **Error (SS within)** | 246 |  | |
| **Error (SS within)** | 234 |  |  |  |  |  |  |

**Significant at the 0.05 level. **Significant at the 0.01 level.*

*DF = Degrees of Freedom, SIG = significance*

***Table 6:*** *ANCOVA Model Results for Carer Data*

| **QCPR follow-up 1 – model 5** | | | | **QoL-AD follow-up 1 – model 6** | | | |
| --- | --- | --- | --- | --- | --- | --- | --- |
| **Factor** | **DF** | **F-value** | **p-value** | **Factor** | **DF** | **F-value** | **p-value** |
| **QCPR carer Baseline** | 1 | 288.79 | **<0.01 | **QoL-AD proxy Baseline** | 1 | 357.58 | **<0.01 |
| **PwD Age** | 1 | 0.76 | 0.39 | **PwD Age** | 1 | 1.06 | 0.30 |
| **Carer Gender** | 1 | 0.57 | 0.45 | **Carer Gender** | 1 | 0.15 | 0.70 |
| **Carer Age** | 1 | 0.20 | 0.66 | **Carer Age** | 1 | 0.03 | 0.87 |
| **PwD Gender** | 1 | 3.16 | 0.08 | **PwD Gender** | 1 | 0.20 | 0.66 |
| **Carer Marital status** | 1 | 5.18 | *0.02 | **Carer Marital status** | 1 | 0.43 | 0.51 |
| **Centre** | 6 | 0.32 | 0.93 | **Centre** | 6 | 0.51 | 0.80 |
| **Wave** | 4 | 0.46 | 0.76 | **Wave** | 4 | 1.26 | 0.29 |
| **Allocation** | 1 | 3.89 | *0.05 | **Allocation** | 1 | 2.10 | 0.15 |
| **Centre x Allocation** | 6 | 1.31 | 0.25 | **Centre x Allocation** | 6 | 2.38 | *0.03 |
| **Researcher Attendance** | 1 | 0.03 | 0.86 | **Researcher Attendance** | 1 | 0.70 | 0.40 |
| **Centre x Researcher Attendance** | 6 | 0.81 | 0.57 | **Centre x Researcher Attendance** | 6 | 0.61 | 0.72 |
| **Error (SS within)** | 270 |  |  | **Error (SS within)** | 285 |  |  |
| **QCPR follow-up 2 – model 7** | | | | **QoL-AD follow-up 2 – model 8** | | | |
| **Factor** | **DF** | **F-value** | **p-value** | **Factor** | **DF** | **F-value** | **p-value** |
| **QCPR carer Baseline** | 1 | 188.6 | **<0.01 | **QoL-AD proxy Baseline** | 1 | 230.61 | **<0.01 |
| **PwD Age** | 1 | 2.22 | 0.14 | **PwD Age** | 1 | 0.29 | 0.59 |
| **Carer Gender** | 1 | 1.27 | 0.26 | **Carer Gender** | 1 | 2.88 | 0.09 |
| **Carer Age** | 1 | 2.71 | 0.10 | **Carer Age** | 1 | 1.14 | 0.29 |
| **PwD Gender** | 1 | 0.25 | 0.62 | **PwD Gender** | 1 | 0.86 | 0.36 |
| **Carer Marital status** | 1 | 0.08 | 0.78 | **Carer Marital status** | 1 | 0.45 | 0.51 |
| **Centre** | 6 | 0.54 | 0.78 | **Centre** | 6 | 1.20 | 0.31 |
| **Wave** | 4 | 0.84 | 0.50 | **Wave** | 4 | 1.12 | 0.35 |
| **Allocation** | 1 | 0.00 | 0.99 | **Allocation** | 1 | 0.23 | 0.63 |
| **Centre x allocation** | 6 | 1.11 | 0.36 | **Centre x Allocation** | 6 | 0.49 | 0.82 |
| **Researcher Attendance** | 2 | 0.46 | 0.63 | **Researcher Attendance** | 2 | 4.15 | *0.02 |
| **Centre x Researcher Attendance** | 10 | 0.98 | 0.46 | **Centre x Researcher Attendance** | 10 | 0.94 | 0.50 |
| **Error (SS within)** | 272 |  | | **PwD age*Researcher Attendance** | 2 | 3.96 | *0.02 |
|  |  |  |  | **Error (SS within)** | 282 |  |  |

**Significant at the 0.05 level. **Significant at the 0.01 level.*

*DF = Degrees of Freedom, SIG = significance*

***Table 7:*** *ANCOVA Model Sensitivity Analysis Results at Follow-up 2*

| **Participant QCPR follow-up 2** | | | | **Participant QoL-AD follow-up 2** | | | |
| --- | --- | --- | --- | --- | --- | --- | --- |
| **Factor** | **DF** | **F-value** | **SIG** | **Factor** | **DF** | **F-value** | **SIG** |
| **QCPR Baseline** | 1 | 47.66 | **<0.01 | **QoL-AD Baseline** | 1 | 91.01 | **<0.01 |
| **Age** | 1 | 5.32 | *0.02 | **Age** | 1 | 0.75 | 0.39 |
| **Gender** | 1 | 0.02 | 0.89 | **Gender** | 1 | 0.22 | 0.64 |
| **Marital status** | 1 | 0.42 | 0.52 | **Marital status** | 1 | 0.56 | 0.46 |
| **Centre** | 6 | 0.84 | 0.54 | **Centre** | 6 | 3.06 | **0.01 |
| **Wave** | 4 | 0.42 | 0.80 | **Wave** | 4 | 1.08 | 0.37 |
| **Allocation** | 1 | 0.56 | 0.45 | **Allocation** | 1 | 0.33 | 0.57 |
| **Centre x Allocation** | 6 | 0.78 | 0.59 | **Centre x Allocation** | 6 | 0.91 | 0.49 |
| **Researcher Attendance** | 2 | 4.02 | *0.02 | **Researcher Attendance** | 2 | 3.32 | *0.04 |
| **Centre x Researcher Attendance** | 11 | 1.53 | 0.12 | **Centre x Researcher Attendance (fu2)** | 11 | 0.89 | 0.55 |
| **Baseline x Researcher Attendance** | 2 | 4.35 | **0.01 | **PwD Age x Researcher Attendance** | 2 | 2.96 | *0.05 |
| **Error (SS within)** | 233 |  |  | **Error (SS within)** | 280 |  |  |
| **Carer QCPR follow-up 2** | | | | **Carer QoL-AD proxy follow-up 2** | | | |
| **Factor** | **DF** | **F-value** | **SIG** | **Factor** | **DF** | **F-value** | **SIG** |
| **QCPR carer Baseline** | 1 | 184.46 | **<0.01 | **QoL-AD proxy Baseline** | 1 | 232.93 | **<0.01 |
| **PwD Age** | 1 | 1.74 | 0.19 | **PwD Age** | 1 | 0.28 | 0.60 |
| **Carer Gender** | 1 | 0.99 | 0.32 | **Carer Gender** | 1 | 3.22 | 0.07 |
| **Carer Age** | 1 | 2.87 | 0.09 | **Carer Age** | 1 | 1.26 | 0.26 |
| **PwD Gender** | 1 | 0.31 | 0.58 | **PwD Gender** | 1 | 1.27 | 0.26 |
| **Carer Marital status** | 1 | 0.06 | 0.81 | **Carer Marital status** | 1 | 0.71 | 0.40 |
| **Centre** | 6 | 0.77 | 0.59 | **Centre** | 6 | 1.29 | 0.26 |
| **Wave** | 4 | 1.34 | 0.26 | **Wave** | 4 | 0.96 | 0.43 |
| **Allocation** | 1 | 0.02 | 0.89 | **Allocation** | 1 | 0.33 | 0.57 |
| **Centre x Allocation** | 6 | 1.08 | 0.37 | **Centre x Allocation** | 6 | 0.55 | 0.77 |
| **Researcher Attendance** | 2 | 1.26 | 0.29 | **Researcher Attendance** | 2 | 4.05 | *0.02 |
| **Centre x Researcher Attendance** | 11 | 0.76 | 0.68 | **Centre x Researcher Attendance** | 11 | 1.12 | 0.35 |
| **Error (SS within)** | 271 |  | | **PwD age x Researcher Attendance** | 2 | 3.98 | *0.02 |
|  |  |  |  | **Error (SS within)** | 281 |  |  |

**Significant at the 0.05 level. **Significant at the 0.01 level.*

*DF = Degrees of Freedom, SIG = significance*

***Table 8:*** *Adjusted Means for Researcher Attendance Groups from PwD ANCOVA Model*

| OUTOME MEASURE | Adjusted values at follow-up 1 | | | | Adjusted mean difference (p – value) | Effect Size  (95% CI) |
| --- | --- | --- | --- | --- | --- | --- |
|  | **Same Researcher** | | **Two Different Researchers** | |  |  |
|  | **N** | **Mean (SE)** | **N** | **Mean (SE)** |  |  |
| QCPR | 132 | 58.0 (0.96) | 144 | 57.9 (1.01) | 0.07  (p = 0.95) | -0.01  (-0.25, 0.23) |
| QoL-AD | 136 | 37.2 (0.64) | 161 | 37.8 (0.72) | -0.66  (p = 0.36) | 0.074  (-0.16, 0.30) |
|  | | | | | | |
| OUTOME MEASURE | **ADJUSTED VALUES AT FOLLOW-UP 2** | | | | | |
|  | **Same Researcher** | | **Two Different Researchers** | | **Three Different Researchers** | |
|  | **N** | **Mean (SE)** | **N** | **Mean (SE)** | **N** | **Mean (SE)** |
| QCPR | 92 | 58.0 (1.10) | 108 | 57.3 (0.86) | 70 | 57.8 (1.34) |
| QoL-AD | 91 | 36.7 (0.87) | 115 | 38.0 (0.68) | 74 | 37.5 (0.97) |

**SE – Standard error**
